# Cardiorenal Syndrome Type 1 in Patients with Heart Failure with Preserved Ejection Fraction

**DOI:** 10.3390/jcm15114033

**Published:** 2026-05-22

**Authors:** Lidija Savic, Ratko Lasica, Gordana Krljanac, Sanja Stankovic, Dragan Matic, Damjan Simic, Lazar Djukanovic, Milika Asanin

**Affiliations:** 1 Faculty of Medicine, University of Belgrade, 11000 Belgrade, Serbia; drlasica@gmail.com (R.L.); grkljanac@gmail.com (G.K.); dragan4m@gmail.com (D.M.);; 2 Cardiology Intensive Care Unit & Cardiology Clinic, Emergency Hospital, University Clinical Center of Serbia, 11000 Belgrade, Serbia; 3 Center for Medical Biochemistry, University Clinical Center of Serbia, 11000 Belgrade, Serbia; sanjastankovic2013@gmail.com; 4 Faculty of Medical Sciences, University of Kragujevac, 34000 Kragujevac, Serbia

**Keywords:** cardiorenal syndrome, acute heart failure with preserved ejection fraction, acute kidney injury, therapy, prognosis

## Abstract

CRS type 1 (CRS-1) is defined as acute kidney injury (AKI) caused by acute decompensated heart failure (ADHF). HF is divided into three subtypes according to the value of ejection fraction (EF). HF with preserved ejection fraction (HFpEF) is an increasingly prevalent subtype of heart failure. A significant number of patients with HFpEF during episodes of acute decompensation (ADHFpEF) develop CRS-1. The objective of this narrative review is to summarize the data about the epidemiology, pathophysiological mechanisms, therapy, and prognostic impact of CRS-1 in patients with ADHFpEF. The most important pathophysiological mechanisms leading to the development of CRS-1 in these patients are hemodynamic disturbances and inflammation. Loop diuretics alone or in combination with other diuretics are the mainstay therapeutic option for treating congestion in patients with CRS-1. Introducing SGLT-2 inhibitors as soon as clinically possible has a positive impact on prognosis. CRS-1 is an independent predictor of a worse outcome in patients with ADHF, although this impact appears to be less associated in patients with HFpEF, than in patients with ADHF with reduced EF. Further studies are needed to better clarify pathophysiological mechanisms and develop treatments that improve cardiorenal outcomes in these patients.

## 1. Introduction

The kidneys and the heart function are closely connected, and the dysfunction or failure of one organ leads to the dysfunction or failure of the other. A spectrum of disorders involving both the heart and kidneys, wherein acute or chronic dysfunction of one organ leads to acute or chronic dysfunction of the other organ, are encompassed by the term cardiorenal syndrome (CRS). There are five types of CRS categorized according to the primarily failing organ (the kidneys or the heart) and according to whether the failure is acute or chronic. CRS type 1 and type 3 are considered as acute, indicating that they are caused by acute deterioration of heart or kidney function, whereas CRS type 2 and type 4 are considered to be chronic conditions as they are caused by chronic heart or kidney failure. Finally, CRS type 5 integrates all cardiac and kidney involvement induced by systemic disease [1,2,3,4,5,6]. The five types of CRS are presented in Table 1.

CRS type 1 (CRS-1) is defined as acute kidney injury (AKI) caused by acute decompensated heart failure (ADHF). AKI is a common complication of ADHF and affects more than 50% of hospitalized patients [7,8,9,10]. AKI is defined by the presence of at least one of the following criteria: increase in serum creatinine level in the range of 0.3–0.5 mg/dL with a decrease in glomerular filtration rate (GFR) range of 9–15 mL/min within 48 h, or a rise in serum creatinine of ≥1.5 times within 7 days, as compared to the baseline value or urine output <0.5 mL/kg/h (e.g., oliguria to anuria) for more than 6 h [11,12,13,14,15,16]. There are four subtypes of CRS-1: (1) de novo ADHF leading to de novo AKI; (2) de novo ADHF leading to AKI in patients with already known chronic kidney disease (CKD); (3) ADHF in patients with previously known chronic heart failure (CHF) leading to de novo AKI; and (4) ADHF in patients with previously known CHF leading to AKI in patients with previously known CKD [15].

Heart failure (HF) is classified into three phenotypes according to left ventricular ejection fraction (EF): HF with reduced EF (HFrEF), mildly reduced EF (HFmrEF), and preserved EF (HFpEF). HFpEF is diagnosed in patients who present with classic heart failure symptoms and signs, along with evidence of left ventricular structural or functional alterations consistent with elevated diastolic pressures and/or increased natriuretic peptide concentrations, while maintaining an EF of 50% or higher [17,18].

HFpEF is an increasingly prevalent subtype of heart failure whose rate has risen in the past decade by 10%, relative to HFrEF. This is caused primarily by the aging of the population and the increasing prevalence of comorbidities, which are also risk factors for HFpEF [17,18].

HFpEF is associated with substantial morbidity, including a HF hospitalization rate of 35% and a 14% mortality rate during two-year follow-up. Compared with patients with HFrEF, patients with HFpEF are generally considered to have lower mortality. The large, MAGGIC meta-analysis has confirmed that the adjusted mortality for patients with HFpEF was considerably lower, as compared with patients with HFrEF, although in many observational studies this difference was negligible [17,18].

More than 50% of patients hospitalized for ADHF have a preserved ejection fraction [19]. According to data from the literature, CRS-1 development during episodes of ADHF seems to be more common in patients with HFpEF, as compared with patients with ADHF who have a reduced ejection fraction (ADHFrEF) [10,18]. CRS significantly complicates the course of in-hospital recovery and affects the prognosis of patients with ADHF. However, most data regarding CRS-1 development, progression, therapeutic options, and prognosis are related to patients with ADHFrEF. There are fewer data about CRS-1 in patients with ADHF and a preserved ejection fraction (ADHFpEF). In fact, most of the data are related to development and prognostic impact of CRS type 2 in these patients [9,20,21,22,23,24]. Since a significant number of patients with ADHF have a preserved EF and are at risk of or develop CRS-1 during hospitalization, it is of clinical importance to understand the mechanisms, the therapeutic approach, and prognostic impact of CRS-1 in patients with ADHFpEF.

The objective of this narrative review is to summarize the data about the epidemiology, specific pathophysiological mechanisms, therapeutic options, and prognostic impact of CRS-1 in patients with ADHFpEF.

## 2. Materials and Methods

A narrative literature review was conducted using the PubMed/MEDLINE database to identify studies evaluating CRS-1 in patients with ADHFpEF. The search was performed for relevant English-language articles published between January 2000 and March 2026 using specific keywords. The search terms included “cardiorenal syndrome,” “acute heart failure,” “heart failure with preserved ejection fraction,” “acute kidney injury,” “pathophysiology,” “prognosis,” and “therapy.”

The initial search identified 198 articles. Following screening, 60 articles were excluded because they did not meet inclusion criteria. Full-text assessment was performed for 138 articles, of which 60 were excluded for reasons including insufficient relevance to CRS-1 or HFpEF, case-report design, or lack of available full text. The reference lists of the included articles were manually examined to identify any further relevant articles. Ultimately, 83 articles were included in the narrative review. Article selection was performed by one author and evaluated/verified by a second author. Evaluation considered factors such as study design, methodology, management of bias and confounding variables, validity and reliability of measurements, statistical analysis, study limitations, and the generalizability of the results.

**Figure 1:**
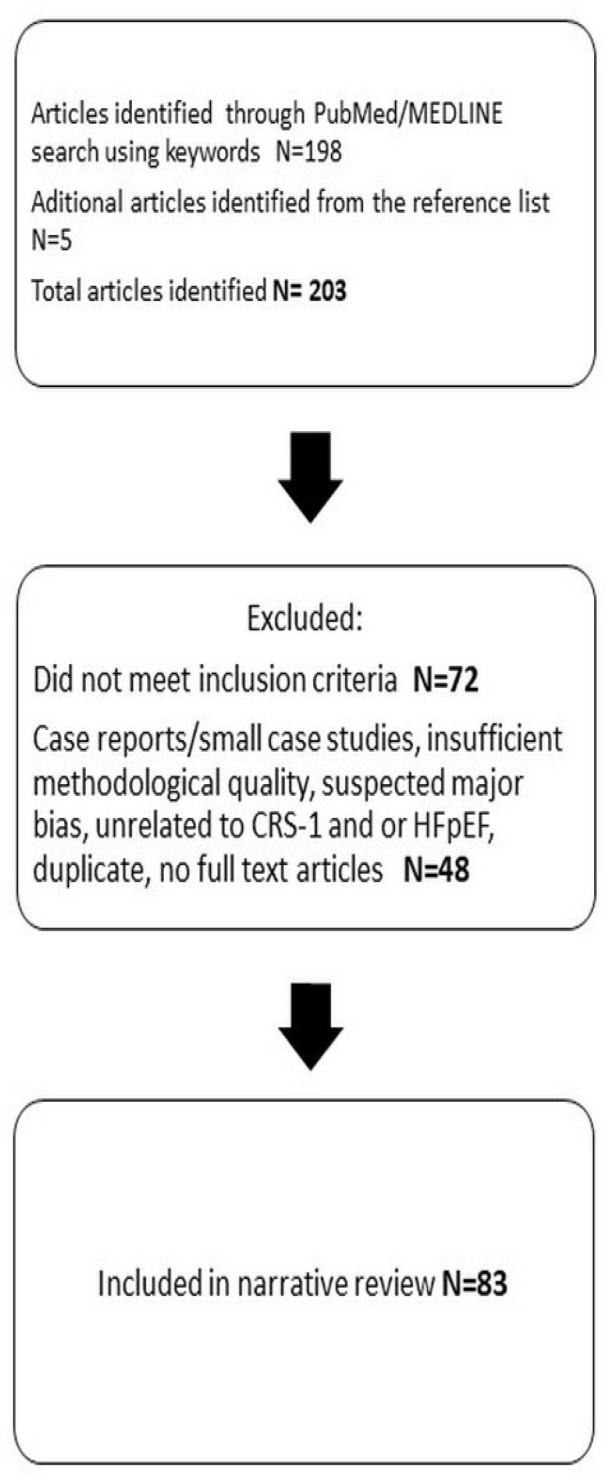
Simplified flow diagram of literature search and study selection process for the narrative review.

Studies were included if they: addressed CRS-1 and/or HFpEF, evaluated pathophysiological mechanisms, diagnosis, biomarkers, management, or outcomes of CRS-1 in HFpEF patients, were randomized controlled trials, observational studies, cohort studies, systematic reviews, meta-analyses, or relevant clinical guidelines and were published in peer-reviewed journals. Studies were excluded if they were case reports or small case series, conference abstracts, non-English publications, studies with insufficient methodological quality or suspected major bias, studies unrelated to CRS-1 or HFpEF, and duplicate publications. Simplified flow diagram of the literature search and study selection process for the narrative review is presented on Figure 1.

Because this study was designed as a narrative review, no original statistical analyses or meta-analytic procedures were performed. Therefore, no statistical software was used.

### 2.1. Risk Factors for CRS-1 Development in Patients with ADHFpEF

Old age, hypertension (HTN), diabetes mellitus (DM), obesity, chronic kidney disease (CKD), and coronary microvascular dysfunction are the most commonly reported risk factors for HFpEF, but also for CRS-1 development in patients during acute decompensation [5,6,19,25,26,27].

Older age (usually ≥60 years) is associated with increased arterial stiffness, myocardial stiffness, decreased diastolic relaxation, and HFpEF development [18,27]. Also, with aging, kidney function declines, which is why older patients have lower GFR values, which places them at higher risk of AKI [27]. Long-standing hypertension (especially if it is poorly controlled) is one of the most common risk factors for HFpEF as it leads to cardiac remodeling. In patients with hypertension, left ventricular remodeling encompasses a spectrum of geometric patterns, including concentric remodeling (the most prevalent form), concentric hypertrophy, and eccentric hypertrophy. These distinct phenotypes reflect different hemodynamic and neurohormonal influences and may have varying implications on diastolic dysfunction and the development of HFpEF [20]. Hypertension is also a risk factor for kidney dysfunction [28]. Furthermore, hypertension at a patient’s initial presentation as a part of ADHF has been associated with CRS-1 development during hospitalization, probably as a reflection of strong neurohumoral activation and sodium and water retention [9,29].

Diabetes mellitus (DM) is one of the most common causes of reduced kidney function and is also a risk factor for myocardial remodeling that leads to HFpEF. Obesity is also a risk factor for HFpEF, but its impact on CRS-1 development is complex and has not been confirmed in all studies. However, many studies have demonstrated that the increased number of adipocytes in obese patients secrete high levels of proinflammatory cytokines, which may, in turn, facilitate the development of AKI in patients with ADHF [5,16,29,30,31,32]. More than 60% of patients with CRS-1 have baseline CKD [2,10]. The explanation for the development of CRS-1 in patients with preserved baseline renal function could be that, during their lifetime, some individuals have repeated episodes of subclinical or clinically unrecognized episodes of AKI. These episodes occur with episodes of dehydration, nephrotoxic therapy for other diseases, etc. With every AKI episode there is injury to some nephron units. Kidneys have the ability to alter their blood flow and filtration and usually recover their function, but the number of remaining nephrons declines. This can be an explanation why some patients with normal renal function easily develop CRS-1 in the settings of ADHF [29]. Therapy for ADHF can also have an impact on CRS-1 development in hospitalized patients—high (loop) diuretic doses may be associated with CRS-1 development probably due to excessive activation of the renin–angiotensin–aldosterone system (RAAS) [16]. Other drugs, such as medication used for DM treatment (e.g., metformin), some antibiotics, or the administration of iodine contrast, may also affect the delicate balance between the heart and the kidneys during episodes of ADHF, and contribute to the occurrence of AKI [29].

Finally, coronary microvascular dysfunction has also been proposed as a novel mechanism for HFpEF and reduced renal function. In the PRevalence Of MIcrovascular DySfunction in Heart Failure with Preserved Ejection Fraction (PROMIS-HFpEF) trial, a worse coronary flow reserve (CFR) was associated with a higher urine albumin-to-creatinine ratio, which is an important diagnostic and prognostic marker in patients with CKD. This finding may indicate that patients with a worse CFR are at higher risk of AKI during an episode of ADHF, but the association of coronary microvascular dysfunction and CRS-1 development should be clarified in future studies [20].

### 2.2. Pathophysiology

The connections between the heart and the kidneys in patients with HFpEF are complex and not as yet completely understood [10,21,26]. The “vicious circle” between the heart and the kidneys that leads to the development and progression of CRS-1 also exists in patients with ADHFpEF, but with some differences, as compared with patients with ADHFrEF. The primary pathophysiological mechanisms leading to the development of AKI and CRS-1 in the context of ADHFpEF are hemodynamic mechanisms and include elevated central venous pressure (CVP), increased intra-abdominal pressure (IAP), and pulmonary hypertension [21,33].

The hallmark of ADHFpEF is the so-called backward heart failure, which causes increased CVP. In animal models, it has been demonstrated that an increase in CVP by 20 mmHg raises pressure in the renal veins, reduces urine flow by 30%, increases interstitial pressure in the kidneys, which leads to compression and increased pressure in the renal tubules and a decline in GFR, and, in the chronic course, also to the development of interstitial fibrosis [20,28,34]. The significance of elevated CVP in the development of CRS was also demonstrated in the clinical Evaluation Study of Congestive Heart Failure and Pulmonary Artery Catheterization Effectiveness (ESCAPE) trial [35]. Elevated IAP (≥8 mmHg) occurs as a consequence of increased CVP and is common in ADHFpEF. Elevated CVP causes a progressive shift of blood from effective arterial blood volume to splanchnic capacitance veins. At some moment, the IAP splanchnic veins capacitance function is overwhelmed, causing a significant rise in IAP (≥12 mmHg). This, in turn, causes the renal venous pressure to rise, causing a drop in GFR and tubular dysfunction, as already mentioned above [20]. Pulmonary hypertension as a consequence of elevated left ventricular filling pressure leading to elevated right atrial pressure also contributes to CVP and the rise in IAP. As a consequence of AKI, neurohumoral activation occurs, leading to further pulmonary vascular remodeling, progression of pulmonary hypertension, and progression of kidney injury [20].

Chronotropic incompetence refers to a failure of the heart rate to rise adequately in response to physical activity. It is frequently seen in patients with heart failure, particularly those with preserved ejection fraction (HFpEF). This condition is associated with autonomic dysfunction, characterized by reduced baroreflex sensitivity and heightened sympathetic activity [20,27,34]. In patients with HFpEF, chronotropic incompetence reflects reduced beta-adrenergic responsiveness and seems to contribute to renal hypoperfusion, which can lead to AKI [20].

All previously mentioned hemodynamic mechanisms lead to the reduction in the gradient in the glomerular capillary network and to a decrease in GFR, which causes an increase in renin activity, an increase in aldosterone levels (generally, activation of the renin–angiotensin–aldosterone system—RAAS), and proteinuria [6,20,27].

RAAS activation is a well-known adaptive mechanism in patients with HFrEF (in whom reduced cardiac output is the first step towards and the main cause of reduced GFR), but it seems that RAAS activation is generally of lesser importance in patients with HFpEF than in patients with HFrEF.

It is well known that systemic inflammatory diseases may significantly contribute to the development of HFpEF. Chronic, poorly controlled inflammation—such as that observed in conditions like rheumatoid arthritis, systemic lupus erythematosus, or inflammatory bowel disease—can promote endothelial dysfunction, increased arterial stiffness, and myocardial fibrosis. Persistent elevation of pro-inflammatory cytokines may further impair diastolic function and exacerbate microvascular dysfunction, thereby increasing the risk of HFpEF development and progression [36]. Endothelial dysfunction and chronic inflammation (through activation of proinflammatory cytokines, such as tumor necrosis factor alpha, interleukin-1, and interleukin-6) seem to be important factors in CRS development in patients with ADHFpEF. Endothelial dysfunction with reduced nitric oxide (NO) bioavailability and signaling is identified as the primary pathophysiological abnormality in patients with HFpEF. Also, NO regulates renal hemodynamic and glomerular microcirculation and inhibits proximal tubular sodium reabsorption. Endothelial dysfunction and reduced NO production leads to disturbances in glomerular circulation and enhances tubular sodium reabsorption [20]. Inflammatory cytokines can induce AKI via activating death signaling receptors which cause tubular cell apoptosis [33].

Other nonhemodynamic factors responsible for the vicious circle between the heart and the kidneys are the reactive oxygen species (ROS). Reactive oxygen species lead to the oxidative injury of cardiomyocytes and renal tubular epithelial cells, thus worsening heart and kidney function [27]. Also, enhanced RAAS activation leads to ROS and stimulates proinflammatory cytokine production with renal consequences, as noted above [6,20,28].

Finally, recently another distinctive phenotype among patients with HFpEF was recognized as heart failure with supranormal ejection fraction (HFsnEF), characterized by left ventricular ejection fraction exceeding the normal range (e.g., EF ≥ 65%). Recent studies suggest that HFsnEF may represent a pathophysiologically distinct entity compared with “classical” HFpEF. Patients with HFsnEF often exhibit a so-called “cold and dry” clinical profile, with relatively less evidence of volume overload and instead features consistent with impaired tissue perfusion, increased vascular stiffness, and systemic inflammation. Patients with HFsnEF have alternative mechanisms leading to CRS-1 development which include reduced effective arterial blood flow, endothelial dysfunction, and more pronounced inflammatory pathways, potentially leading to renal injury in the absence of overt congestion [37,38].

**Figure 2:**
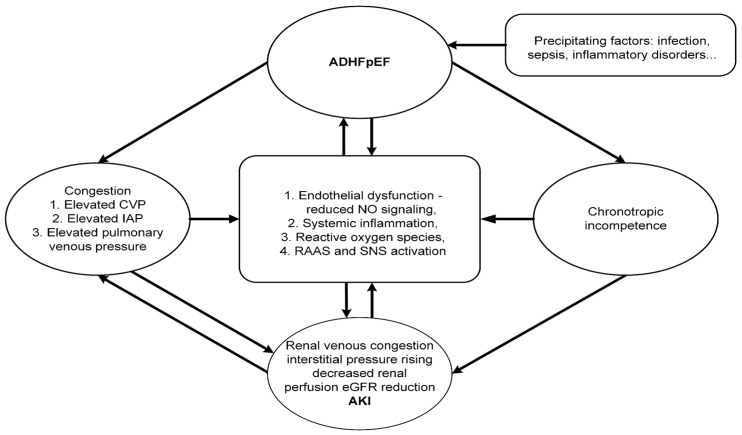
Pathophysiological mechanisms leading to CRS-1 development. CRS-1 = cardiorenal syndrome type 1; CVP = central venous pressure; IAP = intraabdominal pressure; NO = nitric oxide GFR = glomerular filtration rate; RAAS = renin–angiotensin–aldosterone system; SNS = sympathetic nervous system.

The possible mechanisms that lead to CRS-1 development and progression in the setting of ADHFpEF are presented in Figure 2.

## 3. Diagnosis

In patients with acute HFpEF, the CRS-1 diagnosis is established based on the standard criteria for diagnosing ADHFpEF (echocardiographic evidence of preserved EF ≥ 50%, diastolic dysfunction and/or presence of relevant structural heart disease), together with the simultaneous presence of AKI [16,18]. However, diagnosing AKI in patients with ADHF can be complex as small, transitory fluctuations in the serum creatinine level (and urine output) are often observed in patients with ADHF. Diagnosis AKI rests on a combination of clinical assessment of congestion status, relevant hemodynamic parameters, the serum creatinine level, detection of markers of possible intrinsic kidney injury (such as urine microscopy), as well as through the investigation of alternative explanations for serum creatinine elevation [4,8,27].

Echocardiography is the most important method for diagnosing HFpEF. In addition to its use in detailed analysis of cardiac function (systolic and diastolic), echocardiography can be applied to assess central venous pressure, systolic pulmonary artery pressure, pulmonary capillary pressure, and left ventricular filling pressures. Pulmonary ultrasound is important for assessing pulmonary congestion and can potentially serve as a guidance tool for decongestion without triggering the worsening of renal function (WRF), by identifying a reduction in the number of pulmonary B lines [18]. Renal ultrasonography helps assess whether kidney disease is acute or chronic by evaluating features such as kidney size, echogenicity, cortical thickness, and the corticomedullary differentiation. These findings are useful in identifying acute kidney injury (AKI) and detecting evidence of underlying chronic kidney disease (CKD). In addition, Doppler assessment of intrarenal venous flow patterns with ultrasound is an emerging technique for identifying renal venous congestion, which plays a significant role in the development of cardiorenal syndrome type 1 (CRS-1), particularly in patients with ADHFpEF [4,6].

Hemodynamic monitoring provides complementary insights into CRS-1 pathophysiology. Right heart catheterization remains the reference standard and is recommended in selected patients with refractory symptoms or diagnostic uncertainty [18].

Natriuretic peptides are recommended for diagnosis and prognostication, while dynamic markers such as hemoconcentration have been associated with effective decongestion. B-type natriuretic peptide (BNP) and N-terminal-pro BNP (NTproBNP) are well-known biomarkers for myocardial stretch, and are commonly used for diagnosing ADHF [6,18,20], but they are less sensitive for HFpEF [34]. Increased BNP levels during episodes of ADHF are linked to a higher risk of AKI. On the other hand, BNP levels are also significantly higher in patients with CRS-1, as compared with patients with AHF without renal impairment [27]. It should be kept in mind that there are many causes of elevated BNP and NT-proBNP (such as increasing age, atrial fibrillation, acute coronary syndrome, chronic obstructive pulmonary disease, severe infection, etc.) that reduce their diagnostic accuracy. On the other hand, the levels of BNP and NT-proBNP are usually lower in obese patients as compared with non-obese patients [18].

There are many proposed potential biomarkers for diagnosing AKI before a rise in serum creatinine occurs (such as kidney-injury molecules-KIM-a, neutrophil gelatinase-associated lipocalin (NGAL), etc.). However, unfortunately, none of these markers seem reliable for early AKI diagnosis and the follow-up of kidney function, due to their dependence on volume status, renal inflammation, neurohumoral activation, etc. [4,21,38,39].

The most promising biomarker for the early detection of AKI could be NGAL. NGAL (siderocalin) is a biomarker of tubular injury that can be detected in blood and urine in patients with AKI, 48–72 h before the rise in the creatinine level [8,28,33,40]. This biomarker has been studied in CRS, and it seems that it may have some diagnostic and prognostic value in patients with AHF. There are data demonstrating that, in hospitalized patients with ADHF, an elevated plasma NGAL level at admission predicts AKI (although the studied patients were predominantly with ADHFrEF). On the other hand, the value of NGAL in ADHF was analyzed in the AKINESIS trial, which found that plasma NGAL was not superior to creatinine for predicting WRF [6,33]. The urine NGAL level seems to be more sensitive in the early detection of AKI, as compared with the plasma level of NGAL [20]. Its elevated values can be detected in serum even before the level of creatinine begins to rise. One study found that NGAL had a sensitivity of 90% and a specificity of 99% in detecting AKI. One meta analysis that included 10 studies with around 2000 patients with CRS has shown that early serum and urine measurements of NGAL was a predictor of dialysis and death [4].

Another novel biomarker is C-natriuretic peptide (CNP). CNP belongs to the family of natriuretic peptides but demonstrates predominantly antiproliferative and antifibrotic effects. The plasma level of CNP is normally low because CNP predominantly acts as a paracrine and autocrine factor. Urinary CNP is derived from local renal production, and it reflects renal structural integrity and function. Elevated urinary CNP levels have been detected in patients with ADHF, suggesting activation of renal natriuretic peptides in response to acute congestion, and may represent a marker of underlying AKI [6].

Despite all the presented data, currently, the role of novel renal biomarkers applicable for early diagnosis of AKI and CRS-1 needs further validation. In the meantime, serum creatinine-based definitions remain a strong tool in diagnosing AKI in everyday clinical practice [7].

## 4. Therapeutic Options

The most important therapeutic options in patients with ADHFpEF and CRS-1 are presented in Table 2.

Loop diuretics are the mainstay therapeutic option for treating congestion in patients with ADHFpEF and CRS-1 [22,25]. In patients with ADHFpEF, diuretics are absolutely necessary for decongestion, but they must be applied with particular caution to avoid significant reduction in preload during their use, given that patients with HFpEF are preload-sensitive. Any decrease in preload in these patients leads to a reduction in cardiac output, which then further results in renal hypoperfusion and worsening of CRS-1 [20,27].

The fluid volume, fluid distribution, and diuretic response differ in patients with ADHFrEF and ADHFpEF [20]. There are data indicating that patients with ADHFpEF demonstrate an overall lesser intravascular volume expansion and a greater interstitial fluid expansion, as compared with patients with HFrEF. Diuretic therapy in patients with ADHFpEF leads to the loss of more total bodily fluid, as compared with patients with ADHFrEF [41]. In one study, which included patients with ADHF, it was found that the estimated plasma volume reduction with diuretics was associated with more frequent WRF in patients with ADHFpEF, but not in patients with ADHFrEF [20,42]. These findings indicate that patients with ADHFpEF are more susceptible to developing WRF or AKI with diuretic therapy than patients with ADHFrEF, due to the decrease in their preload [20,27].

However, despite these findings, principles of loop diuretics dosing and administration do not differ significantly between patients with ADHFpEF and patients with ADHFrEF. The dosing of diuretics should lead to decongestion, while preserving adequate renal perfusion. Doses of loop diuretics depend on whether patients were on daily oral diuretics before hospital admission or not [18,43,44,45]. Given that HFpEF is common in obese patients, dosing of diuretics in these patients requires higher doses than those that are usually recommended, and dosing according to body weight should also be considered [44].

Although intravenous loop diuretics can be administered as intravenous boluses or as continuous infusion, in the ROPA-DOP study it was found that in patients with ADHFpEF continuous intravenous loop diuretic therapy was associated with a higher rate of AKI, as compared with intermittent intravenous bolus dosing. The explanation for this finding may lie in the preload dependence and different fluid distribution in these patients (as mentioned previously). Continuous intravenous infusion of loop diuretics may not allow the adequate re-equilibration of intra- and extravascular volumes in the setting of acute decongestion, but when using intermittent intravenous boluses there is enough time between boluses for re-equilibration of intra- and extra-vascular volumes [20].

Accurate determination of volume status may be necessary in patients with ADHFpEF, and in patients who do not respond adequately on initial diuretic therapy, right heart catheterization and pulmonary artery catheter implantation should be considered, especially in those with WRF and without adequate diuresis [20]. Also, according to the results of the Pragmatic Urinary Sodium-based treatment algoritHm in Acute Heart Failure (PUSH-AHF) study, natriuresis-guided dosing of diuretic therapy in patients with ADHFpEF (which is based on the same principles as in patients with ADHFrEF) improved diuretic response and alleviated congestion [44,45,46,47,48,49]. These findings were confirmed in a meta-analysis and systematic review performed by Prata et al., which included three randomized controlled trials and two observational studies. Protocolized natriuresis-guided therapy was confirmed safe, reducing the risk of AKI [47]. Target diuresis after starting intravenous loop diuretic therapy in congested patients should be >100–150 mL/h and the spot urine sodium concentration should be >50–70 meq/L [48].

CRS is also defined as a state wherein effects of diuretic therapy are limited by the development of resistance to loop diuretics, which is considered as one of the hallmarks of CRS-1 [6,11,12,28,34,44]. The causes of diuretic resistance in patients with ADHFpEF include enhanced distal nephron sodium reabsorption and elevated CVP, which causes decreased renal perfusion and interstitial congestion that limits loop diuretic delivery to the proximal tubule [44,50]. The most important ways of resolving the problem of diuretic resistance in patients with HFpEF include combinations of diuretics (so-called sequential nephron blockade) and methods of renal replacement therapy (RRT).

The first step in resolving diuretic resistance is the combination of loop diuretics with thiazides and mineralocorticoid receptor antagonists (MRAs). However, it should be noted that studies analyzing the success of diuretic combinations in overcoming resistance have mostly included patients with ADHFrEF. In the Combination of Loop with Thiazide Diuretics in Patients with Decompensated Heart Failure (CLOROTIC) trial, treatment with thiazides in addition to loop diuretics showed greater weight loss in the group with combined therapy. However, the thiazide group showed a slightly higher proportion of patients experiencing WRF compared with those receiving loop diuretics alone [12]. MRAs (spironolactone, eplerenone, and finerenone) are weak diuretics, but in combination with loop diuretics can prevent distal nephron sodium reabsorption, hypokalemia, and in chronic settings can prevent myocardial fibrosis and remodeling [51]. Their long-term positive effect has been demonstrated in many previous studies in patients with HFrEF and in patients with heart failure with middle-range ejection fraction. In a meta-analysis by Zaheen et al. it was shown that in patients with HFpEF treatment with spironolactone and eplerenone improved echocardiographic parameters of diastolic dysfunction and blood pressure control, but had no influence on clinical outcomes [51]. Also, one preclinical study conducted on male hypertensive and diabetic rats with HFpEF revealed that finerenone treatment for 12 weeks resulted in reduced renal hypertrophy and cardiac fibrosis, and also improved cardiac diastolic function and perfusion [52]. MRAs are thought to have positive long-term effects on the kidneys because they are a very important part of neurohumoral blockade. However, in patients with CRS-1 careful titration of MRAs is necessary, with close monitoring of kidney function and electrolytes. There are data indicating that treatment with finerenone was less associated with a rise in serum potassium levels [53,54].

A combination of loop diuretics and acetazolamide was analyzed in the Acetazolamide in Decompensated Heart Failure with Volume Overload (ADVOR) trial. Treatment with acetazolamide also improved diuresis and decongestion, as compared to treatment with loop diuretics only, but with no difference in all-cause mortality and heart failure rehospitalization in the follow-up period [53].

The use of RRT is recommended if, despite all previous measures, there is no effective decongestion. Also, RRT is necessary in patients with CRS-1 and oliguria, electrolyte disbalance, acid–base disbalance, and/or signs of uremia (e.g., patients with AKI and customary nephrological criteria for starting RRT). RRT can result in many adverse events, including further WRF to the terminal stage [48,53]. Unless the patient has uremia, electrolyte disbalance, or metabolic acidosis, in which case starting RRT is necessary as soon as possible, in patients with ongoing congestion and diuretic resistance there is no clear evidence of the best timing for starting RRT [48]. Studies have not demonstrated the superiority of RRT over diuretic therapy, either in terms of decongestion or in terms of prognosis. In a large meta-analysis, early initiation of RRT did not influence 28-days all-cause mortality, as compared with delayed RRT in critically-ill patients with AKI. Furthermore, early initiation of RRT was associated with a higher hypotension rate and with RRT-associated infections [48].

The Diuretic Optimization Strategies Evaluation (DOSE) trial, the Ultrafiltration versus Intravenous Diuretics for Patients Hospitalized for Acute Decompensated Congestive Heart Failure (UNLOAD) trial, and the Cardiorenal Rescue Study in Acute Decompensated Heart Failure (CARRESS-HF) trial evaluated various decongestion approaches in patients with volume overload who did not have AKI [55,56]. In the DOSE trial, worsening renal function (WRF) occurred more frequently in patients receiving high-dose loop diuretics compared with those undergoing ultrafiltration (UF). In contrast, the CARRESS-HF trial found a higher incidence of WRF in patients treated with UF than in those managed with diuretics. UF was also associated with a greater rise in plasma renin activity compared with stepwise pharmacologic therapy [4].

Meanwhile, the UNLOAD trial showed no significant difference in WRF rates between UF and diuretic treatment. Although UF achieved more effective decongestion, it did not translate into improved outcomes compared with diuretics [56]. Overall, findings from CARRESS-HF strongly argue against using UF as a first-line treatment in patients with ADHF [4]. It is very important to note that the above-mentioned trials and meta-analysis comparing UF and diuretic therapy in patients with ADHF did not mention the EF of the included patients (e.g., no differentiation between HFpEF and HFrEF was made) [20].

Other possible ways for overcoming diuretic resistance may include the following: vasodilator therapy, therapy with sodium glucose-2 transporter (SGLT-2) inhibitors, avoiding and correcting hypochloremia, and adding a vasopressin V2 receptor antagonist only in selected patients.

Therapy with direct vasodilators—such as nitrates combined with hydralazine—can modify central hemodynamics and thereby indirectly promote diuresis. However, this approach is generally limited to patients who are hypertensive, and in some cases normotensive, with careful blood pressure monitoring. Although patients with ADHFpEF often have elevated blood pressure, vasodilator therapy, especially venodilators, may cause prompt blood pressure decrease, which decreases pre-load and consequently cardiac output. This may contribute to a significant decrease in renal perfusion and aggravate AKI [55,56,57].

Milrinone acts as an inotropic agent, but with strong pulmonary and systemic vasodilation effects leading to a greater reduction in filling pressures and is often preferred in patients who were previously treated with beta-blockers, as its mechanisms bypass adrenergic signaling [44]. Its effect on lowering filling pressure makes it adequate in certain patients with ADHFpEF. Nevertheless, milrinone should also be used cautiously due to the risk of systemic hypotension [8,44].

It has recently been suggested that the chloride anion is important for preserving serum osmolarity together with sodium. The chloride anion is also responsible for maintaining the fluid acid–base balance, wherein it “competes” with serum bicarbonate. Hypochloremia leads to changes in the distal nephrons, which, in turn, contribute to diuretic resistance and impaired decongestion. Findings from numerous studies have confirmed that there is an association between hypochloremia and poor decongestion [6,50]. However, at this moment, it is unclear whether chloride levels are only markers of severity and prognosis of CRS, or whether they can be a distinctive therapeutic target [6].

Tolvaptan, a selective vasopressin V2 receptor antagonist, has been shown to increase urine output and promote greater weight loss when used alongside standard diuretic therapy, although it has not demonstrated benefits in clinical outcomes or reductions in rehospitalization rates [12]. At present, tolvaptan is primarily indicated for managing marked hypervolemia and euvolemic hyponatremia. It is not approved for the treatment of acute decompensated heart failure (ADHF), even in cases of diuretic resistance, although its use may be considered in patients with ADHF who have hyponatremia that does not respond to ACE inhibitor therapy. Additionally, there is a lack of evidence regarding its effectiveness in ADHF patients across different levels of left ventricular ejection fraction [25].

Currently, it appears that sequential nephron blockade is the most effective method for faster and more efficient decongestion, especially if, after the introduction of loop diuretics, adequate diuresis is not achieved. However, it should be emphasized that the therapeutic approach to the patient must be individualized.

Persistently elevated intraabdominal pressure after therapy has been associated with deterioration of renal function, regardless of central hemodynamic measures [58]. Reduction in intra-abdominal pressure (IAP) following intensive medical therapy seems to be associated with improvement in renal function. Prompt reduction in IAP by paracentesis has been associated with improvement of renal function [59].

In patients with acute HFrEF, there is clear evidence that, even during an episode of acute decompensation, it is advisable to introduce therapy that has proven positive effects on prognosis, or not to discontinue such therapy, if possible. On the other hand, HFpEF is a clinical syndrome with limited therapeutic options [51].

In patients with HFpEF, SGLT-2 inhibitors are the only recommended therapy that has a positive effect on the prognosis and enhances the diuretic effect of loop diuretics in acutely decompensated patients [24,60]. This recommendation is based on the results of the Empagliflozin Outcome Trial in Patients with Chronic Heart Failure with Preserved Ejection Fraction–EMPEROR-Preserved trial, the Dapagliflozin Evaluation to Improve the Lives of Patients with Preserved Ejection Fraction Heart Failure–DELIVER trial, and the DAPA-HF trial. The results of the EMPEROR-Preserved trial have shown beneficial effects of empagliflozin, in terms of the reduction in the combined risk for cardiovascular death or hospitalization for heart failure, in patients with HFpEF, independently of diabetic status. Similar results have been found in the DAPA and DELIVER trials, wherein dapagliflozin was tested [21,34,61,62]. A meta-analysis of EMPEROR and DELIVER trials confirmed a 20% reduction in composite end-point comprising cardiovascular deaths and first hospitalizations for ADHF. The beneficial effects were consistent across the EF range studied. Another meta-analysis, which included data from the DAPA-HF and DELIVER trials, confirmed that the positive effect of dapagliflozin did not differ in relation to EF [61]. SGLT2 inhibitors have positive neurohumoral effects; they also stimulate osmotic diuresis and natriuresis, decrease plasma volume, and lower blood pressure. These effects make them very useful in acute congestion and in patients with diuretic resistance [6,44,62]. When added to loop diuretics, SGLT2 inhibitors can help achieve and maintain euvolemia. Unlike conventional diuretics, SGLT-2 inhibitors preferentially remove interstitial fluid while preserving plasma volume, probably through enhanced lymphatic drainage and by maintaining capillary permeability [24]. In a randomized, blinded study, dapagliflozin initiated early in patients with ADHF improved the diuretic efficiency of loop diuretics [22]. The results of the randomized controlled Dapagliflozin in Acute Heart failure (DICTATE-AHF) trial showed that patients who received dapagliflozin 10 mg were treated with a lower loop diuretic cumulative dose, as compared with patients on the placebo protocol (all patients received loop diuretics with or without metolazone) [63,64]. SGLT-2 inhibitors lead to lower natriuresis compared with thiazide diuretics. One randomized trial has shown that dapagliflozin was not more effective at relieving congestion than metolazone, and patients receiving dapagliflozin received a larger cumulative dose of loop diuretics [48]. On the other hand, the EMPULSE study found that in hemodynamically stable patients with ADHF, introducing empagliflozin within the first three days of hospitalization was associated with clinical benefits defined as: reduced death, reduced number of future ADHF events, longer time to the first ADHF event, and improved symptoms at 90-day follow-up. The results in patients with ADHF were consistent regardless of the EF value [61]. The results of all the abovementioned trials have shown that SGLT-2 inhibitors have a diuretic effect and that they can be safely introduced in hemodynamically stable patients with ADHF [48]. Since SGLT-2 inhibitors also have proven positive effects on renal function, they seem to be a promising treatment in patients with CRS [61]. The initial WRF that may be registered after SGLT-2 introduction is transitory; it is followed by kidney function recovery and has no adverse prognostic impact. A meta-analysis by Neuen et al. has shown a significantly reduced risk of AKI, dialysis, transplantation, and death due to kidney disease in patients treated with SGLT-2 inhibitors vs. placebo [65]. Canagliflozin decreases levels of TNF-alpha, IL-6, matrix metalloproteinases–7, and fibronectin suggesting that this SGLT-2 inhibitor attenuates molecular pathways related to inflammation and fibrosis. These positive mechanisms are important in preventing CKD progression and chronic CRS development, but it remains unclear how much they can reduce CRS-1 development and improve recovery in patients with CRS-1 [6].

The positive effects of RAAS inhibitors and beta-blockers on the prognosis of patients with HFpEF have not been clearly demonstrated. Many patients with HFpEF are treated with RAAS inhibitors due to associated HTN or DM, for which the use of RAAS is indicated. In patients with ADHFpEF and any WRF, introducing or continuing RAAS inhibitors should be done with caution [18,27]. In a meta-analysis that included studies investigating the impact of RAAS inhibitors on WRF in patients with HFrEF and HFpEF (only two studies included patients with HFpEF), it was found that, in patients with HFpEF, RAAS inhibitors induced WRF and these patients had an increased mortality risk [27,66]. The most likely explanation for these findings is that patients with HFpEF are highly preload-dependent, and treatment with ACE inhibitors and sartans can cause vasodilation and hypotension leading to preload reduction. The consequence is a drop in stroke volume and a further decrease in renal blood flow and GFR [20,27,67]. In the PARAMOUNT trial, it was found that sacubitril–valsartan may attenuate the decrease in renal function in patients with HFpEF, as compared with valsartan, in the chronic setting but not in acute HFpEF [67].

In summary, while sodium–glucose cotransporter 2 (SGLT2) inhibitors have emerged as an important component of HF therapy, as supported by trials, treatment strategies for CRS-1 in patients with HFpEF remain largely supportive and continue to rely on optimization of volume status and hemodynamics. In this context, diuretic therapy remains the cornerstone, yet diuretic resistance is a frequent and clinically significant challenge. Strategies endorsed by the current guidelines include dose escalation, sequential nephron blockade (e.g., addition of thiazide-type diuretics), and careful monitoring of response; however, robust trial evidence guiding these approaches remains limited, and they are often associated with electrolyte disturbances and worsening renal function [18].

**Figure 3:**
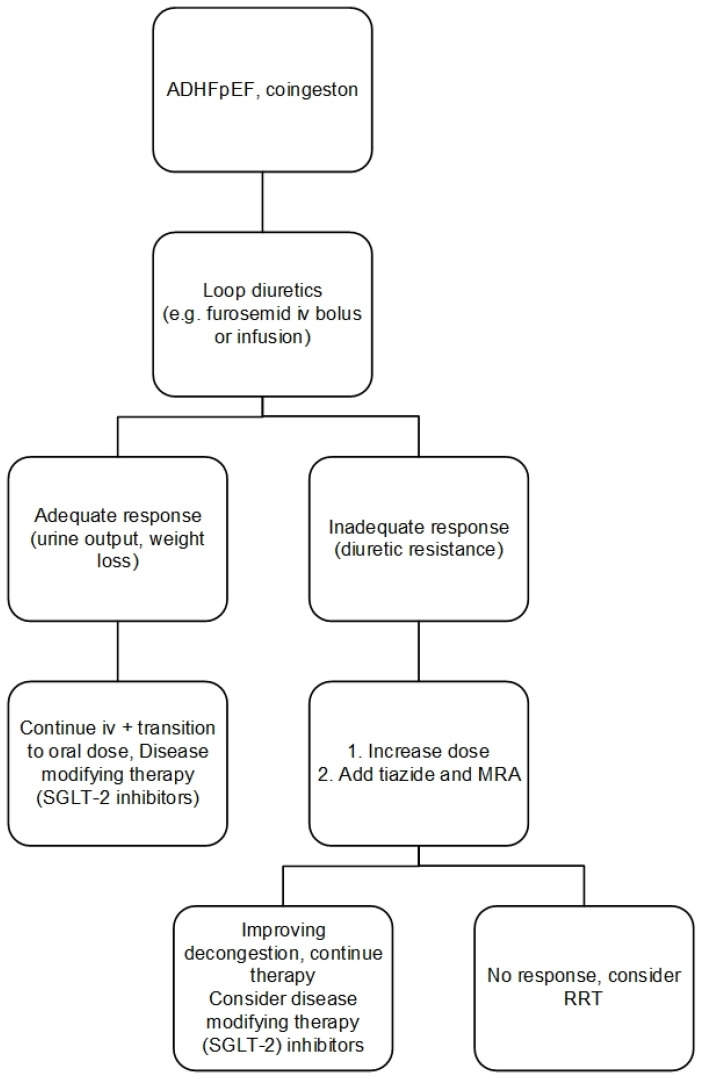
A simple algorithm for a therapeutic approach in patients with ADHFpEF and CRS-1. ADHFpEF = acute decompensated heart failure with preserved ejection fraction; MRA = mineralocorticosteroid receptor antagonist; SGLT-2 = sodium–glucose contrasporter-2; RRT = renal replacement therapy.

In Figure 3, we present a simple algorithm for a therapeutic approach in patients with ADHFpEF and CRS-1.

Finally, clinicians should always search for precipitating conditions that can cause ADHF and affect renal function, e.g., sepsis, systemic inflammatory disorders, etc. [34]. All conditions that precipitate ADHF and can also aggravate renal function should be identified in a timely fashion and properly treated. At the same time, medications that can adversely influence renal function (such as non-steroid anti-inflammatory drugs) should be discontinued [28].

The difference between transitory worsening of renal function (WRF) and CRS-1, and their prognostic impact.

During the natural course of cardiac dysfunction, cardiorenal interaction is of critical importance as reduced renal function predicts cardiovascular mortality and other adverse events [10,23,28,33,68]. Both AKI and CKD are well-known independent predictors of a worse outcome in patients with heart failure, although this negative impact appears to be less associated with patients with HFpEF, as compared to patients with HFrEF [18]. Exceptions to this rule are patients with ADHF and transitory WRF, but with signs of decongestion (e.g., weight loss, decreasing natriuretic peptides, relief of symptoms); data show that they do not have an increased mortality risk nor a higher risk of other adverse events. Conversely, progressive renal dysfunction in the absence of adequate decongestion—or accompanied by signs of hypoperfusion—should raise concern for true CRS-1. The different prognostic impact of WRF and CRS-1 was also confirmed in a study by Sharma et al. where it was found that WRF in ADHFpEF with successful decongestion had no impact on one-year survival and hospital readmission [9]. Therefore, transitory WRF is not associated with poor prognosis in patients with ADHF, as long as decongestion is accomplished [69]. WRF, alongside clinical improvement and decongestion, is more frequently observed in patients with preserved renal function and should not be interpreted to favor one diuretic strategy over another [62], nor should it be considered as an early marker of CRS development. In patients with ongoing congestion, a rising creatinine level is considered as a marker of AKI/CRS-1 development, which is associated with a worse outcome, as compared with patients with ADHF without AKI [69]. Biomarkers may further aid in differentiation between transitory WRF and true AKI: markers of tubular injury (e.g., NGAL, KIM-1) and persistent elevation of NP or inflammatory markers may suggest structural kidney injury and ongoing cardiorenal interaction. On the other hand, isolated creatinine rise with hemoconcentration and with lowering serum NP level reflect a hemodynamic effect and transitory WRF, but not true AKI [6,18,57,69,70,71,72,73].

The occurrence of CRS-1 is associated not only with adverse cardiovascular outcomes; affected patients are more likely to progress to end-stage kidney disease or experience further renal function deterioration [8].

As previously mentioned, the largest amount of data from the literature on the prognostic significance of AKI/CRS-1 relates to patients with HFrEF [27]. In a smaller number of studies that included patients with ADHFpEF, CRS-1 was associated with a more frequent need for hemodynamic support during hospitalization, longer in-hospital stay and higher in-hospital 30-day, 3-month, and 5-month mortality, as compared with ADHFpEF patients with stable kidney function [8,69,70]. The renal arterial resistive index (RI) is a Doppler index of renal blood flow. RI has recently been identified as a potential new marker of renal vascular and parenchymal abnormalities and an indicator of prognosis in patients with HFpEF. Increased RI is a marker of adverse prognosis in patients with HFpEF, even in those with normal GFR, and its prognostic value is additive to the prognostic value of lower eGFR [21].

Finally, patients who develop CRS-1 more often have higher creatinine levels at hospital discharge, as compared with patients who do not develop CRS-1. Also, most patients who develop CRS-1 do not achieve baseline creatinine levels, i.e., they develop some stage of CKD [10]. This was confirmed in a study which included patients with ADHFpEF. CRS-1 development was associated with an increased need for dialysis during hospitalization and lower rates of renal recovery by discharge. Nearly half of the analyzed patients with CRS-1 experienced deterioration in renal function during follow-up, along with a higher readmission rate for ADHF. Lasting kidney damage that remains after an episode of acute kidney injury (AKI) puts patients at increased risk of cardiovascular events and all-cause mortality, both in the short term and over longer follow-up periods [8].

## 5. Future Directions

There are numerous studies investigating novel agents for treating HFpEF and improving renal function. Although, at this moment, none of the tested approaches have shown significant clinical benefit, it is important to mention the direction of further research. Some of the newly tested agents act through nitric oxide (NO) signaling and include serelaxin and inorganic nitrates. Serelaxin was the first agent to be tested. It is a recombinant human seralexin-2 hormone that promotes NO production through stimulation of widely distributed relaxin/insulin-like family peptide receptor 1 (RXFP1) and causes vasodilation. However, it increases renal blood flow and reduces pulmonary artery and wedge pressure. In RELAX-AHF-2, there was no difference in cardiovascular death, worsening of heart failure, and renal failure at day 5, as compared to the placebo, in patients hospitalized for AHF. However, in a biomarker substudy of RELAX-AHF2, serelaxin significantly reduced the worsening of ADHF through day 5 and decreased plasma concentrations of cardiac, renal and hepatic injury biomarkers. Most of these biomarkers return to the baseline value soon after intravenous serelaxin is discontinued. It has therefore been suggested that similar agents acting through NO pathways, but with a longer half-life and administered for a longer period of time, might provide better clinical outcomes in patients with HFpEF [74,75,76]. Several long-acting agents are currently being developed [75]. Inorganic nitrates represent another group of agents acting through NO signaling. They restore NO cyclic guanosine monophosphate (cGMP) signaling and potentially attenuate diastolic dysfunction, pulmonary vascular disease, and endothelial dysfunction [20]. Experimental studies have demonstrated that geranylgeranylacetone (GGA) can increase nitric oxide (NO) activity, reduce myocardial stiffness, and upregulate heat shock proteins 1 and 5 (HSPB1 and HSPB5). In healthy individuals, it also appears to improve endothelial NO activity via enhanced HSP90 expression. In both acute and chronic models of kidney injury, GGA has been shown to stimulate renal HSP70 production, lessen tubular damage, and help prevent worsening renal function. However, findings from the GLADIATOR-HFpEF trial did not demonstrate any significant benefit in echocardiographic indices of diastolic or endothelial function, exercise performance, or renal function in patients with HFpEF [77].

Other agents showing promise are monoclonal antibodies that target inflammatory cytokines and the innate and humoral immune system to combat inflammation and improve endothelial function [5,12,78]. In the context of CRS-1 in patients with HFpEF, anti-inflammatory biological therapies are being explored as a way to interrupt the bidirectional inflammatory signaling between the heart and kidneys that contributes to acute decompensation. Agents targeting key cytokines—such as interleukin-1 (IL-1) inhibitors (e.g., anakinra) or interleukin-6 (IL-6) pathway blockers (e.g., tocilizumab)—have shown potential in reducing systemic inflammation, improving endothelial function, and possibly attenuating congestion-related organ injury. In HFpEF, where chronic low-grade inflammation and microvascular dysfunction play central roles, these therapies may help stabilize hemodynamics and limit renal impairment during acute episodes. However, evidence specifically in CRS-1 remains limited, and further clinical trials are needed to clarify their safety, efficacy, and optimal patient selection [79].

Finally, novel circulatory renal assist devices for the treatment of CRS-1 are being developed with the aim to improve renal arterial perfusion and reduce renal venous congestion [80,81].

Using implantable hemodynamic monitoring with a pressure sensor implanted in the pulmonary artery branch to monitor right-sided pressures was associated with reduction of cardiac decompensation and HF hospitalization [82]. Interatrial shunt devices are used for the treatment of increased left atrial pressure in patients with HFpEF. These devices have potential roles in CRS if they can reduce the rate of HFpEF acute decompensation [83].

However, further studies are needed to determine the efficacy and long-term benefits of these novel devices and therapeutic procedures.

### Study Limitations

This narrative review has several limitations. First, only the PubMed/MEDLINE database was searched, which may have resulted in the omission of relevant studies indexed in other databases such as Embase, Scopus, or Web of Science. Second, the narrative review design may introduce selection bias compared with systematic reviews or meta-analyses. Third, heterogeneity among included studies regarding patient populations limited direct comparison of findings. Finally, only English-language publications were included.

## 6. Conclusions

The number of patients with HFpEF is rising, and they are at risk of CRS development, especially during episodes of acute decompensation. Development of CRS-1 in patients with ADHFpEF complicates the treatment strategy and significantly affects short- and long-term outcomes in these patients. Knowledge and understanding of specific hemodynamic disturbances in patients with HFpEF that lead to renal dysfunction and the development of CRS-1 is necessary in order to adapt the therapeutic approach with the aim of achieving decongestion while preserving renal perfusion. Introducing SGLT-2 inhibitors as soon as clinically possible can have a positive impact on reducing cardiorenal adverse events. Even with progress in diagnostic and treatment approaches, care for CRS-1 in patients with ADHFpEF is still mainly supportive and often derived from evidence in broader heart failure groups. The diverse nature of HFpEF suggests that the underlying mechanisms of CRS-1—and the most effective treatments—are unlikely to be the same for every patient within this population. Further studies are needed to achieve a better understanding of pathophysiological mechanisms, to validate the use of new biomarkers for early diagnosis and prognosis, and to introduce new treatment protocols, which would have a positive impact not only on cardiac, but also on renal outcomes in these patients.

## Figures and Tables

**Table 1:** Classification of cardiorenal syndrome.

CRS Type	Name	Etiology/Primarily Failing Organ
1	Acute cardiorenal syndrome	Acute decompensated heart failure (ADHF)/cardiogenic shock; acute myocardial infarction (AMI); acute myocarditis, etc.
2	Chronic cardiorenal syndrome	Chronic heart failure (CHF) resulting in chronic kidney disease (CKD)
3	Acute renocardiac syndrome	Acute kidney injury (AKI) resulting in HF
4	Chronic renocardiac syndrome	Chronic kidney disease (CKD) resulting in CKD-associated cardiomyopathy and heart failure (HF)
5	Secondary cardiorenal syndrome	Systemic condition/disease causing simultaneous kidney and heart dysfunction/failure (e.g., amyloidosis, sepsis, cirrhosis)

**Table 2:** The most important therapeutic options in patients with ADHFpEF and CRS-1.

Therapy	Comments
Loop diuretics	- The mainstay of therapy for decongestion - Can be used alone or in combination with other diuretics as a part of sequential nephron blockade - Can cause enhanced RAAS activation and WRF/AKI
Mineralocorticosteroid receptor antagonists (MRAs)	- Successful in combination with loop diuretics, as a part of sequential nephron blockade - May have positive effects on the heart and kidneys in chronic settings - Caution: hyperkalemia
SGLT2 inhibitors	- In the acute setting: may enhance diuresis when combined with loop diuretics - In the chronic setting: disease-modifying agents that improve prognosis in patients with HFpEF - Initial WRF is typically temporary, followed by recovery of kidney function, and does not carry negative prognostic significance.
ACEi inhibitors/ARB/ARNI	- No prognostic impact in patients with HFpEF - Used for treatment of concomitant conditions HTN, DM, etc. - Cautions: vasodilation and reduced preload may further cause a decline in kidney function.
Renal replacement therapy (RRT)	- If all previous measures do not lead to effective decongestion; mandatory in patients with volume overload, oliguria, electrolyte and/or metabolic acid–base disbalance

HFpEF = heart failure with preserved ejection fraction; CRS-1 = cardiorenal syndrome type 1; SGLT-2 = sodium–glucose cotransporter; WRF = worsening of renal function; ACEis = angiotensin converting enzyme inhibitors; ARB = angiotensin receptor blocker; ARNI = angiotensin receptor blocker, neprilysin inhibitor.

## Data Availability

No new data were created or analyzed in this study. Data sharing is not applicable to this article.
